# Female urethroplasty with dorsal onlay buccal mucosal graft: a single institution experience

**DOI:** 10.1007/s11255-023-03520-5

**Published:** 2023-03-01

**Authors:** Margaret M. Higgins, Derek Wengryn, David Koslov, Janine Oliver, Brian J. Flynn

**Affiliations:** Division of Urology, University of Colorado Anschutz Medical Campus, 12631 East 17th Avenue, C-319, Aurora, CO 80045 USA

**Keywords:** Female urethral stricture disease, Urethroplasty, Buccal mucosal graft

## Abstract

**Purpose:**

Female urethral stricture disease is frequently unrecognized or misdiagnosed, with controversy in the literature regarding the definition of strictures and approach to management. The purpose of this study is to report our institutional experience with female urethroplasty and add our experience to the growing body of research.

**Methods:**

We performed a retrospective review of patients undergoing female urethroplasty with dorsal onlay BMG at the University of Colorado between March 2015 and December 2021 performed by two surgeons (BF and JO). The primary outcome measure was surgical success, defined as no stricture recurrence. The secondary outcome measure was the incidence of de novo urinary incontinence.

**Results:**

23 patients were included in our data analysis. The median duration of lower urinary tract symptoms prior to urethroplasty was 16 years. 87% had undergone previous dilations. At a median follow-up of 12.2 months (range 1–81 months), four patients required a secondary procedure for obstruction with an overall success rate of 83%. One patient developed de novo stress urinary incontinence and one patient developed urge urinary incontinence. Subgroup analysis was performed comparing the patients that developed stricture recurrence (*N* = 4) to those that did not (*N* = 19). Those with stricture recurrence had a longer duration of symptoms and more dilations prior to urethroplasty.

**Conclusion:**

Female urethroplasty with BMG is effective at treating female urethral stricture disease, with excellent outcomes at over a year of follow-up and minimal risk of stress incontinence postoperatively.

## Introduction

Female urethral stricture disease (FUSD) is an uncommon pathology affecting between 4 and 13% of women presenting with bladder outlet obstruction, which is estimated to affect 3–8% of women [1–3]. It is difficult to diagnose due to lack of consensus on definition as well as the variability in normal female voiding parameters and presenting symptoms [2]. One of the more concise definitions for FUSD is “a symptomatic anatomical narrowing of the female urethra based on direct visualization and/or radiological evidence and/or urethral calibration with the exclusion of other competing etiologies” [3].

Once the diagnosis is made, dilations are usually the initial treatment, despite poor long-term efficacy [1]. Conversely, in male urethral stricture disease (MUSD), the urologist is more inclined to recommend urethroplasty due to well-recognized, durable success. Urethroplasty adoption in women has been slower due to lack of consensus on surgical approach and graft material, as well as concerns for complications such as de novo incontinence and fistula. Multiple urethroplasty techniques have been described utilizing flaps or grafts. Most of the literature is small case series or descriptive studies.

Using our expertise with male urethroplasty, we extrapolated our preferred dorsal onlay buccal mucosa grafting (BMG) technique to female urethroplasty. Multiple case series have demonstrated success with this technique; however, they have had small sample sizes. Our aim was to examine our outcomes and add our experience to the growing body of research.

## Methods

After obtaining IRB exempt status approval (COMIRB 13-1444), we performed a retrospective review of patients undergoing female urethroplasty with dorsal onlay BMG at the University of Colorado and Denver Health hospitals between March 2015 and December 2021. These were performed by two surgeons (BF and JO). Patient demographics, surgical variables and post-operative outcomes were collected. The primary outcome measure was surgical success, defined as no stricture recurrence. Stricture recurrence was defined by cystoscopy. The secondary outcome measure was the incidence of de novo urinary incontinence. We also compared pre- and post-operative uroflowmetry parameters if available.

Descriptive analyses were carried out for all patient characteristics of interest. We then compared two groups (stricture recurrence vs non-recurrence). Quantiles (median, minimum, maximum, 1st and 3rd quartiles) were computed and graphically displayed in boxplot form.

### Surgical technique

**Fig. 1:**
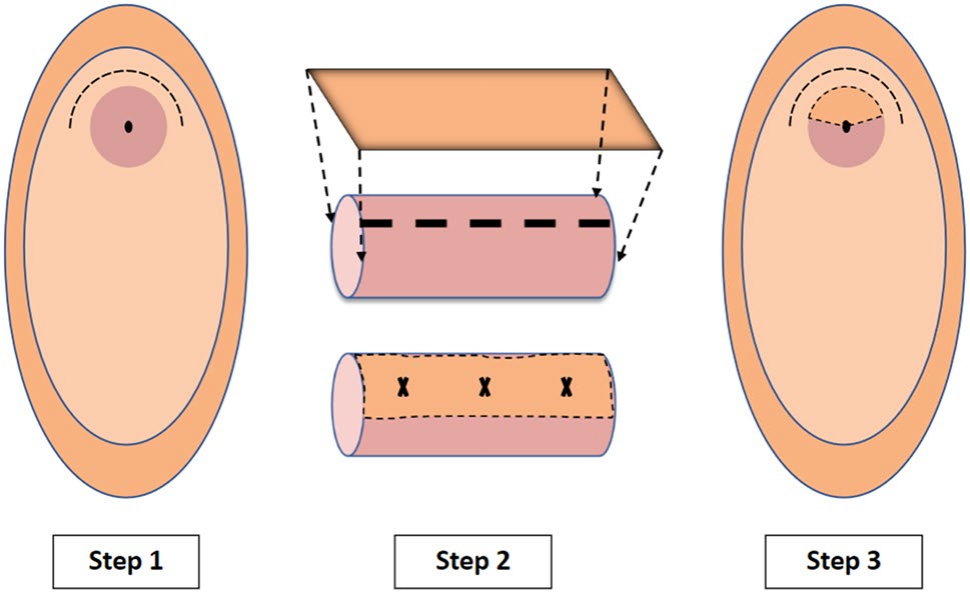
Aschematic demonstrating our surgical technique. Step 1: Inverted U incision is made around the meatus. The urethra is dissected off the anterior vaginal wall. Step 2: A dorsal urethrotomy is made and a buccal mucosal graft is inlayed and secured with PDS suture. Step 3: Vaginal incision closed and 14Fr urethral foley left for 2 weeks

Surgical technique was similar for both surgeons (Fig. 1). The patient is placed in lithotomy position. Cystoscopy and suprapubic catheter (SPC) placement are performed. A 5-French open-ended catheter is placed per urethra. A dorsal curvilinear incision from 10 to 2 o’clock is made. The urethra is then detached from its dorsal attachments, sparing the pubourethral ligaments laterally and clitoral cavernosal tissue dorsally. The urethra is then incised at the 12 o’clock position, intermittently placing stay sutures to allow for more proximal visualization. The mucosa and sponge are carefully dissected and incised proximally, progressing toward the bladder neck until the stricture has been completely incised. Cystourethroscopy and/or urethral calibration with bougies à boule may be performed at this stage to ensure incision has been carried out beyond the stricture. The apical sutures are placed using 4–0 polydioxanone (PDS).

The BMG is then harvested off the buccinator muscle from the cheek, ensuring a graft length to match the urethral incision with a goal width of 2.5 cm the graft is then de-fatted on the back table and mouth is packed with epinephrine-soaked gauze.

The graft is then brought into the surgical field and sewn into place dorsally with the previously placed 4–0 PDS sutures. Several tacking sutures are placed to secure the graft to the dorsal tissue bed. Additional 4–0 PDS sutures are then placed along the lateral urethral edges to re-tubularize the urethra to the graft. The urethral meatus is then re-approximated to the surrounding vaginal and vestibular epithelium with 4–0 poliglecaprone 25. We then perform a final cystoscopy to confirm patency of the repair. A 14 Fr urethral foley is left in place capped and an 18-French SPC is attached to drainage.

The foley catheter is removed at 2 weeks and a voiding trial commences. Once post-void residuals are low, the SPC is removed. All patients undergo post-operative assessment of lower urinary tract symptoms and post-void residual. Imaging is not routinely performed. If any concerning symptoms develop, an office cystoscopy was performed using a 17-French rigid cystoscope.

## Results

A total of 23 patients underwent dorsal onlay BMG urethroplasty and were included in our data analysis. The median age was 50. Etiology was primarily idiopathic (Table 1).

**Table 1:** Patient characteristics and outcomes

Characteristic	Cohort (*N* = 23)
Age, years	50 (34–84)
BMI	30 (21–38)
Stricture etiology	
Idiopathic	19 (83%)
Iatrogenic	4 (17%)
Prior stricture-related procedure	20 (87%)
Self-dilation	9 (39%)
Stricture length, cm	3 (1.5–4)
Catheter time, days	13 (1–36)
Follow-up, months	12.2 (1–81)

Median (range) or incidence (percentage)

Pre-operative stricture work-up included cystoscopy (*N* = 23), uroflowmetry (*N* = 8), voiding cystourethrogram (*N* = 3), and/or urodynamics (*N* = 7). The most common presenting symptoms included storage lower urinary tract symptoms in 15 (65%), voiding lower urinary tract symptoms in 12 (52%), and recurrent urinary tract infections in 10 (44%). The median duration from onset of uncharacterized lower urinary tract symptoms to the time of urethroplasty was 16 years. Moreover, there was a median of seven years from the onset of symptoms to the time of urethral stricture diagnosis s (range 3–19 years). Twenty patients (87%) had undergone previous dilations, with a median of three operative dilations (range 1–20).

At a median follow-up of 12.2 months (range 1–81 months), four patients required a secondary procedure (urethral dilation or DVIU) for obstruction with an overall success rate of 83%. Of the patients that developed recurrences, all had had previous dilations, one patient had had previous vaginal flap urethroplasty, and another had concomitant urethrovaginal fistula repair. Median time to recurrence was 3.8 months (range 1–65 months). Of the patients with greater than one year follow-up (*N* = 12), three developed stricture recurrence. One patient developed de novo stress urinary incontinence and underwent an autologous fascia pubovaginal sling. One patient developed de novo urge urinary incontinence which was managed with sacral neuromodulation. Neither of the patients had undergone pre-operative urodynamics. Two patients had acute graft donor-site complaints that resolved. One patient had buccal contracture that was managed conservatively. No patient developed a urethrovaginal fistula. No patient reported de novo dyspareunia either.

The median post-operative maximal flow (*Q*_max_) on uroflowmetry was 15.9 mL/s (6.6–26 mL/s, *N* = 9) compared to median pre-operative *Q*_max_ 10.8 mL/s (2–18.2 mL/s, *N* = 8). Of the patients that had pre- and post-operative post-void residual recorded (*N* = 20), the median improvement was 15 cc. Of those that had both pre- and post-op uroflowmetry evaluations (*N* = 5), the median improvement of *Q*_max_ was 8.2 mL/sec.

**Fig. 2:**
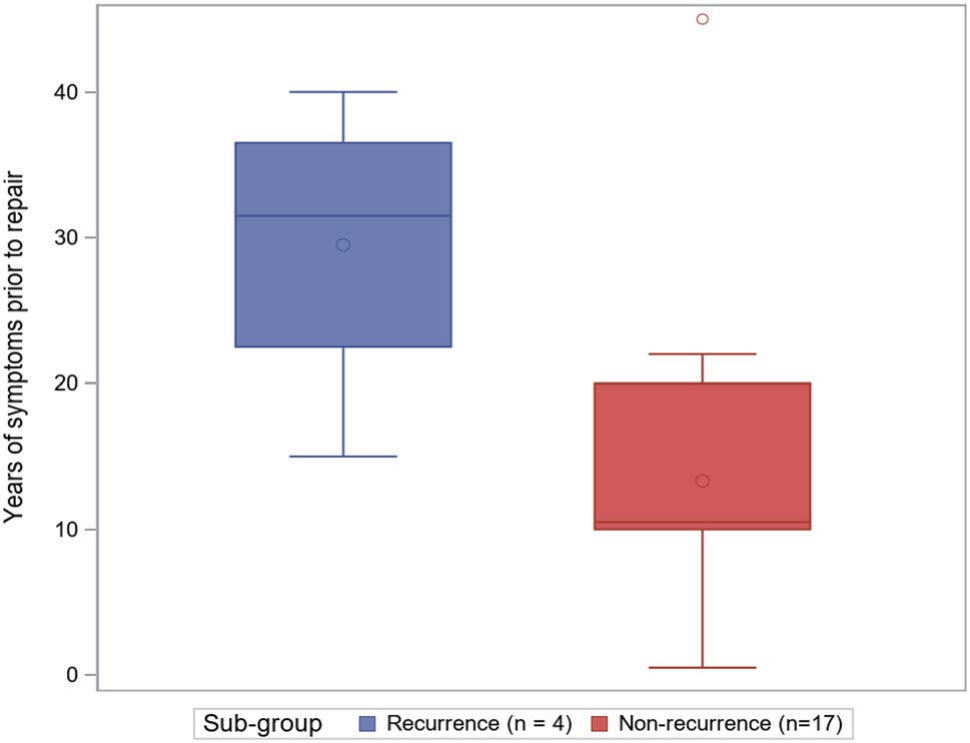
Boxplot graph comparing years of symptoms prior to urethroplasty of patients that developed stricture recurrence (blue) versus those that did not (red)

**Fig. 3:**
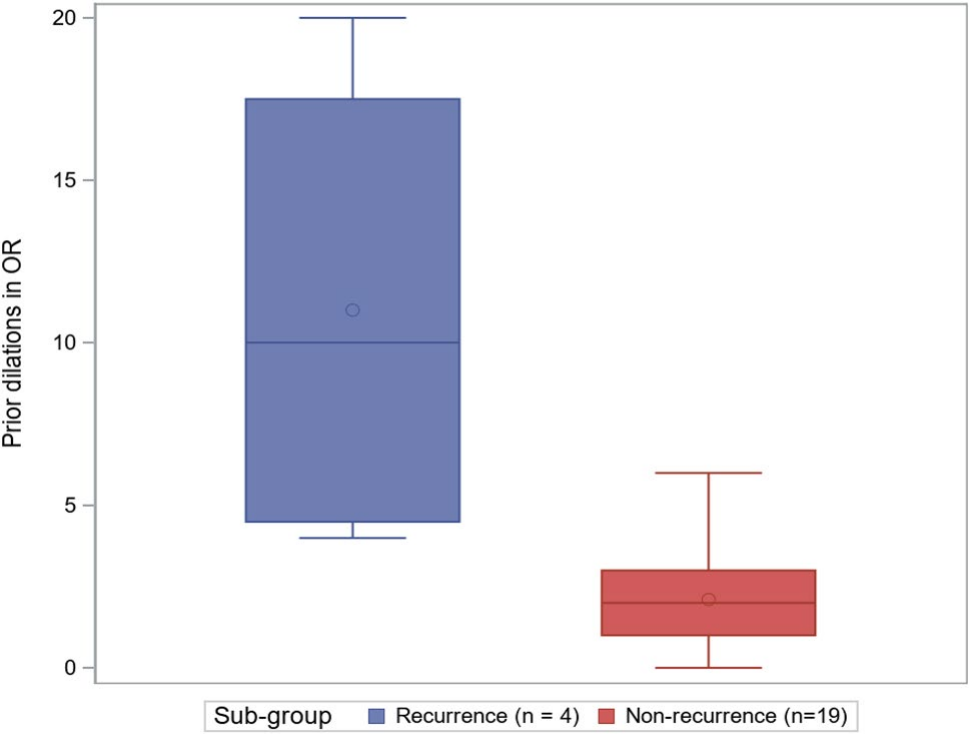
Boxplot graph comparing the numbers of dilations performed in the operating room (OR) prior to urethroplasty of patients that developed stricture recurrence (blue) versus those that did not (red)

Subgroup analysis was performed comparing the patients that developed stricture recurrence (*N* = 4) to those that did not (*N* = 19) (Table 2). While our small sample size is underpowered to find statistical significance, those with stricture recurrence had a longer duration of symptoms (Fig. 2) and more dilations prior to urethroplasty (Fig. 3).

**Table 2:** Subgroup analysis comparing the patients that developed urethral stricture recurrence to those that did not have a recurrence

	Non-recurrence						Recurrence					
	*N*	Median	Min	Max	Q1	Q3	*N*	Median	Min	Max	Q1	Q3
Age, years	19	54.0	31.0	84.0	40.0	61.0	4	47.5	38.0	55.0	42.0	52.0
BMI	19	29.9	21.4	37.5	23.2	32.0	4	28.1	25.3	33.6	26.7	30.9
Stricture length, cm	15	3.0	1.5	4.0	2.5	3.5	4	2.8	2.0	3.5	2.3	3.3
Stricture caliber, Fr	18	9.0	1.0	16.0	5.0	14.0	4	9.0	3.0	16.0	4.5	14.0
Years of symptoms prior to repair	17	10.5	0.5	45.0	10.0	20.0	4	31.5	15.0	40.0	22.5	36.5
Prior dilations in OR	19	2.0	0.0	6.0	1.0	3.0	4	10.0	4.0	20.0	4.5	17.5
Urethral foley duration, days	19	13.0	6.0	36.0	10.0	20.0	4	10.5	1.0	29.0	4.5	21.0
Follow-up, months	19	7.2	1.0	82.0	4.1	30.4	4	10.5	4.3	67.7	4.8	41.7

## Discussion

FUSD can be difficult to diagnose and can affect women for many years prior to proper diagnosis and definitive repair. Endoscopic management is most commonly the first treatment given the technical ease and relative low risk of dilation; however, this has not been shown to hold long-term symptom relief [3]. Our series adds to the growing body of data on patients with FUSD (Table 3) [4–9]. Dorsal BMG urethroplasty is successful in treating FUSD with low complication rate. Our study also highlights a known gap in success rates of female BMG urethroplasty compared to male urethroplasty. In our cohort, there was a median of 16 years between diagnosis and urethroplasty and seven years from onset of symptoms to diagnosis of FUSD which highlights the prolonged symptomatic course for many of these patients. This potential delay in treatment is not well quantified in the literature. One multi-institutional retrospective study found a median of 1 year (IQR 0–6 years) between diagnosis of FUSD and surgery, 2 years (0–10 years) specifically for urethroplasty with free graft [10]. Another study cites mean time from onset of symptoms to urethroplasty was 44 months (8–340 months) [9]. Most studies do not quantify the time from diagnosis to urethroplasty, but the number of prior treatments is frequently reported.

**Table 3:** Literature review of success rates of dorsal onlay BMG female urethroplasty in series with > 15 subjects

Study	Number of subjects	Stricture free rate	Mean follow-up (months)
Sharma et al. [5]	15	93%	12
Hampson et al. [7]	39	77%	32
Khawaja et al. [6]	25	92%	25.5
Kore et al. [7]	21	90%	25
Richard et al. [8]	19	91%	12 (median)
Gomez et al. [9]	17 (15 without irradiated pts)	76% (87% if excluding irradiated pts)	15
Current study	23	83%	23

There have been multiple case series describing repair techniques and outcomes, including several that use our preferred method of dorsal onlay BMG. Richard et al. retrospectively reviewed the outcome in 19 patients and demonstrated a clinical success rate of 94.7% at 1–3 months and 90.9% at 1 year with an incidence of de novo SUI in only 9% [8]. Success was primarily defined as any subjective improvement on the patient global impression of improvement (PGI-I). Khawaja et al. in a prospective trial of 25 patients that underwent dorsal BMG urethroplasty reported a (92%) success with no incontinence at an average follow-up of 25.5 months. [6] Hampson et al. published a multi-institutional retrospective experience of 39 dorsal BMG urethroplasties performed by 6 high-volume reconstructive surgeons over a 10 year period [7]. Average follow-up was 33 months and recurrence rate was 23%.

Extrapolated from the experience in MUSD, urethroplasty with BMG in FUSD has been shown to have durable success. Our recurrence rate (diagnosed by cystoscopy) of 17% and de novo incontinence rate of 8.7% is consistent with the literature [2, 4, 11]. We prefer using buccal as compared to vaginal or labial flaps for its ease of harvesting, ready availability, no visible scar, rich intradermal plexus, and to preserve vaginal tissue. Though the ventral approach has the benefit of familiarity, we prefer dorsal approach for several reasons. First, it maintains the ventrolateral urethral support structures (pubourethral ligaments, lateral urethropelvic ligaments, laterosuperior fixation to the arcus tendineous) [9]. Grafting to the firm dorsal tissues reduces risk of sacculation and vaginal fistula. Also, for non-meatal sparing strictures, ventral onlay creates a ventral, often patulous neomeatus. Only one randomized study has looked at a head-to-head comparison between ventral and dorsal BMG female urethroplasty. Though limited by small sample size (nine in each group) and short follow-up (6 months), they found comparable success rates between the two techniques noting preference for dorsal technique for distal strictures and ventral for more proximal strictures [12]. No fistula, sexual dysfunction, or incontinence were reported for either group.

In our series, patients with recurrent stricture had increased number of dilations prior to their urethroplasty. In MUSD, the limited effectiveness of dilation is well established [13]. Cure rates around 50% after the first procedure precipitously drop with subsequent endoscopic treatments [14]. Similar to the trend found in our cohort, multiple endoscopic interventions have been shown to reduce efficacy of urethroplasty in MUSD [15]. Primary urethroplasty is becoming more common in MUSD, however, FUSD is often first treated with dilation (87% of our cohort). This, despite a lack of scientific evidence supporting the long-term efficacy. There have been a handful of randomized controlled trials of varying strengths evaluating the success of urethral dilation, but none of these addressed FUSD specifically [16]. Instead, they were targeting recurrent urinary tract infections or non-specific lower urinary tract symptoms.

With the more extensive literature and clinical definitions in MUSD, how does the presentation compare to FUSD presentation? In one study evaluating bulbar urethroplasty in men, only 6% (24 out of 363 patients) had not had a urethral procedure prior to urethroplasty [17]. This is similar to our cohort with only 13% having no previous dilations, and is comparable to the literature [7]. The number of interventions might be higher in FUSD. In our cohort, the median number of dilations prior to urethroplasty was three (range 0–20). In one large study of 363 men with MUSD, 65% had had prior DVIU and 55% prior dilation, but only 18% and 43% had had multiple DVIUs or dilations respectively. [17] Like in FUSD, male patients also present in a heterogenous way. While retention, difficult catheterization, gross hematuria, and recurrent UTIs occur for some, many present with storage LUTS. One study of MUSD, found the most prevalent pre-operative symptoms were urinary frequency, urgency and nocturia with rates of 52.5, 48.8 and 41.6% respectively. [18]

Limitations of our study include a small sample size and limited follow-up similar to other contemporary studies, and being underpowered to allow statistical comparisons. In terms of follow-up, though we had a broad range, including our more recent cases brought down our follow-up (median 12.2 months and mean 23 months). It has been shown that mean time to recurrence was 14.4 months. [7] An additional limitation in our study, similar to the literature on FUSD, is non-uniform work-up, standardized follow-up and patient reported outcomes such as the incidence of dyspareunia. There are theoretical sexual side effects following urethroplasty that are being examined in MUSD [19]. This is rarely reported in FUSD studies. There is one prospective study that did focus on patient reported sexual side effects using the Female Sexual Function Inventory (FSFI) score both preoperatively and at 3 months following dorsal onlay vaginal graft urethroplasty [20]. They surprisingly demonstrated a 6.4 point improvement in the FSFI scores. More research is needed to determine sexual side effects of FUSD reconstruction.

## Conclusion

Our series adds to the growing body of data on dorsal BMG urethroplasty in women with female urethral stricture disease. Dorsal BMG urethroplasty is successful in treating FUSD with low recurrence rates and rare complication such as de novo incontinence or urethrovaginal fistula in short term follow-up.
